# Heterologous Expression of Xylanase xAor from *Aspergillus* *oryzae* in *Komagataella phaffii* T07

**DOI:** 10.3390/ijms23158741

**Published:** 2022-08-05

**Authors:** Andrey Valentinovich Zadorozhny, Viktor Sergeevich Ushakov, Alexei Sergeevich Rozanov, Natalia Vladimirovna Bogacheva, Valeria Nikolayevna Shlyakhtun, Mikhail Evgenyevich Voskoboev, Anton Vladimirovich Korzhuk, Vladislav Anatolevich Romancev, Svetlana Valerevna Bannikova, Irina Anatolyevna Mescheryakova, Egor Vladimirovich Antonov, Asya Rifhatovna Vasilieva, Elena Iurevna Pavlova, Danil Olegovich Chesnokov, Elizaveta Dmitrievna Shedko, Alla Viktorovna Bryanskaya, Denis Vladimirovich Bochkov, Tatiana Nikolayevna Goryachkovskaya, Sergey Evgenyevich Peltek

**Affiliations:** 1 Laboratory of Molecular Biotechnology, The Institute of Cytology and Genetics, SB RAS, 630090 Novosibirsk, Russia; 2 Kurchatov Genomic Center of the Institute of Cytology and Genetics, SB RAS, 630090 Novosibirsk, Russia; 3 EFKO (Efirnoe Joint-Stock Company), Belgorod Region, 309817 Alekseyevka, Russia

**Keywords:** xylanase, *Komagatella phaffii*, genetic engineering, bioreactor, producer strain

## Abstract

Xylanases (EC 3.2.1.8) hydrolyze the hemicellulose of plant cell walls. Xylanases are used in the food and paper industries and for bioconversion of lignocellulose to biofuel. In this work, the producer-strain with four copies of the xAor xylanase gene was organized in two tandem copies for optimal expression in *Komagataella phaffii* T07 yeast. The secreted 35 kDa xylanase was purified from culture medium by gel filtration on Sephadex G-25 and anion exchange chromatography on DEAE-Sepharose 6HF. Tryptic peptides of the recombinant enzyme were analyzed by liquid chromatography-tandem mass spectrometry where the amino acid sequence corresponded to Protein Accession # O94163 for Endo-1,4-beta-xylanase from *Aspergillus oryzae* RIB40. The recombinant xylanase was produced in a bioreactor where the secreted enzyme hydrolyzed oat xylane with an activity of 258240 IU/mL. High activity in the culture medium suggested xylanase could be used for industrial applications without being purified or concentrated. The pH optimum for xylanase *xAor* was 7.5, though the enzyme was active from pH 2.5 to pH 10. Xylanase was active at temperatures from 35 °C to 85 °C with a maximum at 60 °C. In conclusion, this protocol yields soluble, secreted xylanase suitable for industrial scale production.

## 1. Introduction

Xylanase are related to diverse groups of enzymes, hydrolyzing the main hemicellulose of plant cell walls—xylanes [1]. Xylanes are amongst the dominant compounds of lignocellulase biomass. Mechanism of xylanases is random cleavage of β-1,4-connected D-xylopyranose monomers, composing xylane carbon skeleton homopolymer structure. Nowadays xylanases are combined in glycoside hydrolase families (GH) 5, 7, 8, 10, 11, 26, 30, and 43, with the best studied mechanisms of 10th and 11th families [2].

In this work, gene of endo-1,4-xylanase F1 of *Aspergillus oryzae* was chosen for further cloning and heterologous expression. *Aspergillus oryzae* is one of the most common multicellular fungi in biotechnology for having highly active enzymes with wide substrate spectrum; among them proteases, cellulases, amilases, pektinases, and β-galactosidases are the most notable [3]. Xylanase *xAor* belongs to GH10 family. The characteristic of GH10 family is the presence of TIM barrel (α/β)_8_, being a typical trait of class A enzymes. Active site contains conservative glycone subsites with high substrate affinity and less conservative sites with lower affinity. Glycone subsites from −2 to −1 and from −3 to −1 bind strongly to xylobiose and xylotriose, respectively. All the substrates, exceeding this size, are hydrolyzed from non-reducing end after three xylose monomers, with the presence of two consecutive non-substituted xylose monomers being essential for GH10 family [4].

Hemicellulases, such as xylanases, are the most important enzymes group with broad industrial application, including bioconversion lignocellulose in biofuel, SCP (single-cell protein), bio-whitening, fabric production, juices clarification, sewage purification, and ink-washing [5]. Application in all abovementioned fields reduces usage of dangerous chemical compounds leading to lower toxic load on environment [6].

Various genus *Aspergillus* fungi have an ability to decompose xylans [7]. Chemical structures of xylans vary depending on the source of various xylanases with various hydrolytic activities, physicochemical properties, and structures decomposing them, with endoxylanases being the most important ones [8]. Xylanases are used to decompose lignocellulose to obtain various additives, such as flavor compounds vanillin and guaiacol, or food sweeteners, such as xylitol. In animal feed, xylanases are used to obtain arabino-xylooligosaccharides which are reported to have prebiotic properties. Xylanases are also used in bioconversion to bioalcohols. In pulp and paper industry, traditional bleaching process involves using aggressive chemicals, such as chlorine dioxide, sodium hydroxide, hydrogen, and chlorine peroxides, while application of xylanases provides less environmental damage. It is also important to note that products of hydrolysis of xylan hydrolyses such as ferulic acid and other hydroxycinnamic are used in pharmacological industry [9]. 

Notwithstanding, it is of great importance that in most cases production of xylanases in host fungi is impeded by the low generation velocity and aeration complications in viscous media [10]. Concerning the actuality of recombinant proteins production, it is crucial to obtain producer strains with higher yields of target protein than in natural hosts. 

In our laboratory, producer strain was constructed carrying four copies of xylanase *xAor* gene under control of AOX1 gene promoter and terminator with subsequent cultivation in bioreactor.

## 2. Results

### 2.1. Construction of Genetic Construction for Expression in K. phaffii T07

*Komagataella phaffii* T07 strain deposed in National bioresource center in All-Russian collection of industrial microorganisms (VKPM) based in Kurchatov Institute Research Center (Accession Number #Y-4936) (Appendix A). Optimized sequence of xylanase xAor consisted of 981 bp. Plasmid *pPZL-4*x*-*OA*-xyl-AsOr-* carrying four copies of the xylanase gene of *A. oryzae* under the control of promoter and terminator of the AOX1 gene for inductive expression in *K. phaffii*, was obtained throughout the course of work. Plasmid *pPZL-4*x*-*OA*-xyl-AsOr-* also contains replication origin for expression in *E. coli* and zeocin resistance gene under control of EM7 promoter for *E. coli* expression and TEF1 for *K. phaffii* expression. After transformation, strain of *Komagataella phaffii T07 4*x*-*OA*-xyl-AsOr-* (Accession Number *#*Y-5026 in All-Russian collection of industrial microorganisms (VKPM)) was obtained (Appendix A).

### 2.2. Activity Conditions of xAor Xylanase

Xylanase *xAor* activity was calculated based on xylose calibration, wherein molar extinction coefficient was shown to be ε ≈ 0.00045 ± 0.00005 µMol ^−1^ × cm^−1^. It was shown, that at pH = 7.5, enzyme activity was E = 258,240 IU/mL for culture liquid, containing a total of 3.1 g of xylanase. 

Specific activity for culture liquid was shown to be 37.23 × 10^6^ IU/(mg protein). For lyophilized cultural liquid after tangential centrifugation specific activity was 57.69 × 10^6^ IU/(mg protein) with total amount of 1.8 g. Lyophilized xylanase after chromatographic purification showed activity of 129.53 × 10^6^ IU/(mg protein) and has a total amount of 0.32 g of protein. 

**Figure 1:**
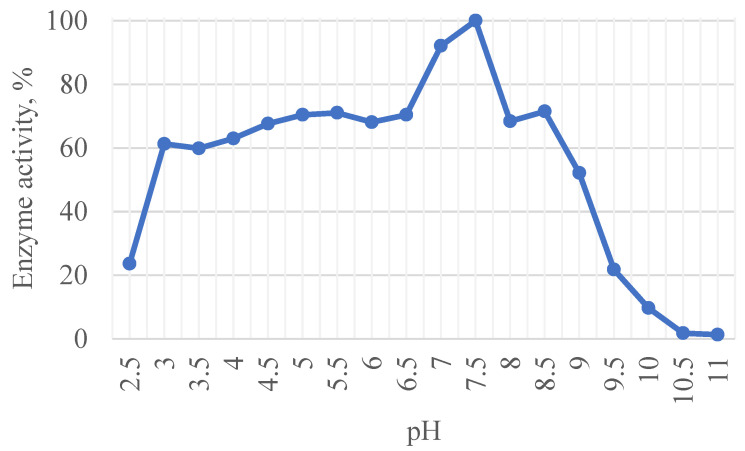
pH optimum for xylanase xAor.

**Figure 2:**
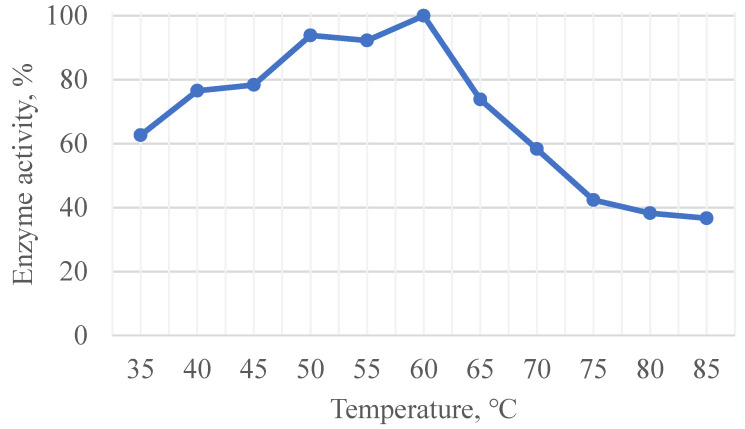
Temperature optimum for xylanase xAor.

pH-optimum of xylanase was 7.5, while working range was shown to be from pH = 3.0 to pH = 9.0, with still showing activity at pH = 2.5 and pH = 10.0 (Figure 1). The enzyme was irreversibly inactivated below pH 2.5 and above pH 10. Subsequent transfer to pH 7.5 buffer did not restore enzyme activity. Temperature optimum was shown to be 60 °C, although it was shown that enzyme is still highly active in temperatures up to 85 °C (Figure 2). Incubating enzyme solution with 0.002 mg/mL at 85 °C and pH = 7.5 for 12 h it was shown that enzyme retain 82% of its initial activity, for 24 h—75%, and for 72 h—57% of activity.

### 2.3. Electrophoretic Analysis and Mass-Spectrometry

**Figure 3:**
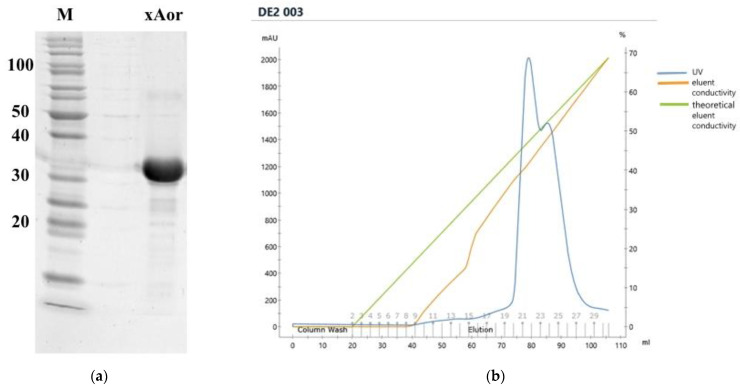
(**a**) Electrophoretic analysis of xylanase xAor, M—molecular mass ladder PageRuler Unstained Protein Ladder (ThermoScientific, Waltham, MA, USA). (**b**) Chromatographic purification of xylanase xAor, performed with anionite DEAE-Sepharose 6HF (Biotoolomics, Great Britain).

Nominal mass of recombinant xAor was 35,552 Da, and matched calculated mass—35.41 kDa. Purity of studied sample was verified with electrophoretic analysis (Figure 3a,b). Isoelectric point was calculated with instrument software 4.73.

**Figure 4:**
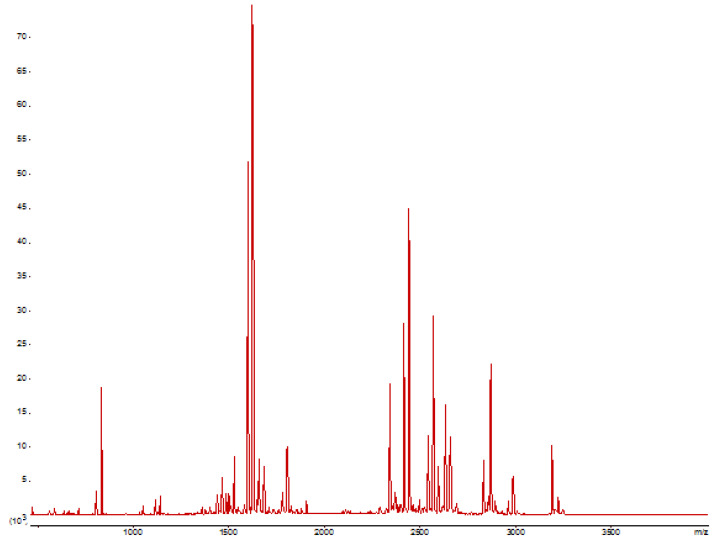

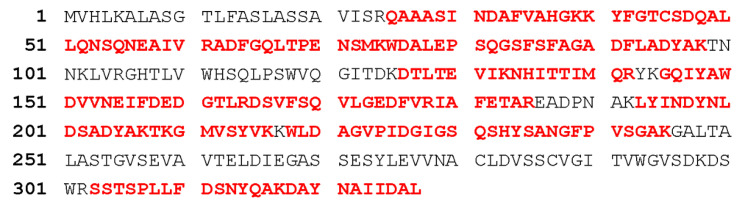
Mass-spectrometry MS1 analysis (verified amino acids of xAor are highlighted red) (Protein accession number: O94163 Endo-1,4-beta-xylanase Aspergillus oryzae RIB40).

PS (protein score) was shown to be 1492, which is statistically significant result. MS sequence coverage was 63% (Figure 4). To determine the protein, SwissProt database was used [11].

Thus, mass-spectrometry of the studied sample showed the identity of the primary target sequence compared to the predicted one (*p* ≥ 0.95).

### 2.4. Xylanase xAor Cultivation in Bioreactor

**Figure 5:**
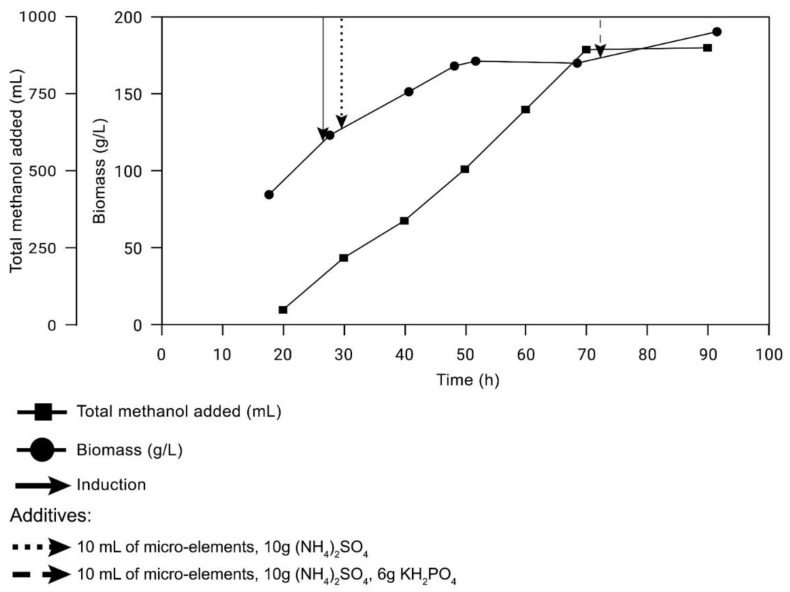
Condition of xAor growth in bioreactor.

Upon reaching 90 g/L biomass [12] and oxygen level shift in bioreactor [13] with 5 L of a yeast culture in salt media, the culture was inducted with the aliquot of 60% methanol and also 10 mL of microelements and 10 g of (NH_4_)_2_SO_4_. The induction stage began with 40 mL of methanol and lowering the temperature down to 27 °C. After the adaption of the culture to methanol and reduction of oxygen, periodical methanol replenishment was started, wherein replenishment was carried out every 20 min in the first three hours, and by the end of the cultivation process volume of added methanol increased twice. It is important to highlight that wherein abrupt increase of oxygen higher than 25% was shown, methanol was added. Periodically, samples were collected to monitor specific enzyme yield and biomass (Figure 5). Cultivation was conducted for three days after the induction. 

In conclusion, obtaining the producer strain of *xAor* xylanase and verifying its ability to be cultivated in bioreactor allow authors to suggest that it may be successfully used to scale the production of *xAor* xylanase at industrial level. Moreover, the authors imply that high activity of the enzyme both in terms of pH and temperature range may be of great value for industrial application of the enzyme.

## 3. Discussion

*Komagataella phaffii* (formerly known as *Pichia pastoris*) is a methylotrophic yeast widely used in biotechnological industry due to its high secretory capacity [14] and ability to grow over 100 g/L on methanol [12]. Moreover, it is of great importance to reduce the effects of heterologous expression, such as posttranslational modifications [15]. Xylanase *xAor* was obtained from *A. oryzae* fungi, thus, expression in fungal organism is more preferable for correct processing of the target protein. Taking into consideration the abovementioned, strain *K. phaffii* T07 (Accession Number #Y-4936), previously discovered in our laboratory, was shown to be the most suitable candidate as a base for producer strain of xAor xylanase. 

For the first time heterologous expression of 10th family xylanase Aoxyn10 from *A. oryzae* in *Pichia pastoris* was described in Yin et al.’s work [16]. Gene AoXyn10 consisted of 2308 bp, including cDNA was 1689 bp, 5′,3′-non-translating region, and 1422 bp. open read frame. In this work, xylanase gene *xAor* from *A. oryzae*, consisted of 981 bp. after the deletion of signal peptides. xAor gene was inserted into four tandem copies since it was shown that multicopy plasmids with recombinant DNA tandem clusters are effective for increasing production of target protein in case of *K. phaffii* transformation [17]. Extracellularly expressed Aoxyn10 activity was shown to be 45 IU/mL [16], while activity of xylanase xAor was 258,240 IU/mL in culture liquid. 

It was shown that fungi xylanases have higher enzyme activity, comparing with bacterial ones, with most of them active at 50 °C and pH range from 4 to 6 [18]. Described in the work of He et al., xylanases from *Aspergillus oryzae* HML366 XynH1 are identical to *A. oryzae* RIB40 protein XP_001826985.1 of glycosyl hydrolases family 10, with the optimal specific activity being 476.9 U/mg at pH 6.0, while remaining active within pH 4.0–10.0 and at a temperature below 70 °C [19]. For *Aspergillus oryzae* F1 endo-1,4-xylanase, it was shown that pH optimum is 5.0, and temperature optimum is 60 °C [20]. Extracellularly expressed Aoxyn10, pH optimum 5.5, and temperature optimum 60 °C, nonetheless xylanase was stable in pH range from 4.0 to 7.0, and temperatures lower than 50 °C [16]. Bhardwaj et al. [5] showed that xylanase XynF1 from *A. oryzae* LC1 in expression system *E. coli* BL21 (DE3) had specific enzyme activity 1037.3 EU/mg, being 9.3 times higher, than in host organism. Recombinant xylanase XynF1 [5] with molecular weight of 37 kDa shows specific enzyme activity in pH spectrum from 3.0 to 10.0, and from 30 °C to 70 °C, wherein pH and temperature optimum are 5.0 and 30 °C, respectively. Thus, xylanase xAor pH working range from 3.0 to 9.0, and temperature range up to 85 °C, which is comparable with other known xylanases.

In conclusion, in this work, soluble, secreted xylanase *xAor* was obtained. The ability of xAor producer strains to be grown in bioreactors and high specific activity of *xAor* xylanase even in culture liquid suggest that the strain obtained is suitable for industrial scale production.

## 4. Materials and Methods

### 4.1. The Constructing of xAor Gene

After GenBank database analysis, sequence of xynF1 gene from *Aspergillus oryzae* (strain ATCC 42149/RIB 40) was chosen (Sequence ID: O94163). Codon composition was calculated according to the table of codons occurrence frequency, optimal for expression in *K. phaffii.* Signal peptides, detected with using algorithm SignalP-5.0 [21], were removed. Genetic constructions were synthetized by Atg:biosynthetics (Merzhausen, Germany), further inserted in primary genetic construction using Gibson assay [22]. Colonies screening for the inserted DNA was performed using PCR method. Amplification was carried out using next protocol—95 °C 3 min; 35 cycles: 95 °C 10 s, 62 °C 15 s 72 °C 30 s. To select a clone with target genetic construction, obtained amplicons were separated electrophoretically and visualized. 

### 4.2. Construction of Multicopy Plasmid

For the construction of the plasmid with two tandem genetic construction copies, expression construction target fragment was amplified from the primary plasmid. Amplification was performed according to the next—95 °C 3 min; 5 cycles: 95 °C 10 s, 58 °C 15 s, 72 °C 80 s; 20 cycles: 95 °C 10 s, 63 °C 15 s 72 °C 80 s. Amplicon, purified with magnetic beads, was treated with restriction enzymes *Mal*I, *Bam*HI, and *Bgl*II at 37 °C for 30 min. Plasmid *pPZL-ost-xyl-Aor*, containing one xAor gene copy, was hydrolyzed with *Bgl*II restriction enzyme at 37 °C for 30 min, and then with alkaline phosphatase 16 °C for 120 min. Obtained DNA fragments with sticky ends were purified using KAPA Pure Beads (Roche, Basel, Switzerland), according to the manufacturer’s instructions, and were used in ligation reaction.

Ligation was carried out using highly active T7 DNA ligase (SibEnzyme, Novosibirsk, Russian Federation) at 16 °C for 30 min. Ligation reaction was purified with magnetic beads, as described above. Next, competent *E. coli* strain XL-blue cells were electoporated with obtained solution, after suspending in 1 mL of LB media and incubating for 45 min at 37 °C. 1/100 of cells were spread on agarized LB media with the addition of 20 µg/mL zeocin and incubated at 37 °C all night.

To confirm the correct orientation of the second expression construction, obtained colonies were incubated in 4 mL of LB media with 20 µg/mL concentration of zeocin all night at 37 °C and 250 rpm. Next, plasmid DNA was extracted using QIAprep Spin Miniprep Kit (Qiagen, Germantown, MD, USA), according to manufacturer’s guidelines. Aliquots of extracted plasmid DNA were treated with restriction enzymes *Bgl*II and *Bam*HI. Then, the presence of the second copy and accuracy of orientation were confirmed by electrophoresis in 1% agarose gel. For the construction of the plasmid with four copies of expression construction, abovementioned assay was performed twice. 

To sum up, plasmid carrying xAor gene under control of AOX1 promoter and terminator for inductive expression in *K. phaffii* yeasts, plasmid replication origin, and zeocin resistance gen under control of EM7 promoter for *E. coli* expression and TEF1 promoter for *K. phaffii* expression, was obtained. 

### 4.3. Plasmid Insertion in Genome of K. phaffii T07

Plasmid *pPZL-4*x*-*OA*-xyl-AsOr-* was electoporated into yeast cells, after transformants were cultivated for 2 h 30 °C in 1 mL 1 M sorbitol. Next, 1/5 of obtained cell suspension was spread on 1.5% agarized BD Difco media (BD, Great Britain) with the addition of 200 µg/mL zeocin.

### 4.4. Evaluation of Enzyme Activity of Obtained Strains

The presence of xylanase expression was verified by inoculation of the obtained colonies in 24 deep well plate, wherein every colony was inoculated in separate well, containing 2 mL 1% yeast extract, 2% peptone with 0.3% glucose and 1% methanol (YPGM). Incubation was carried out at 30 °C, while no pH level observation was performed. *K. phaffii* T07 strain without the inserted xylanase gene was used as a negative control. Plate was placed in thermo-shaker at 360 rpm, wherein every 24 h 200 µL 10% methanol was added to every well. On the third day of cultivation, aliquots were taken and centrifuged at 4000× *g* for 5 min to precipitate cells.

Enzyme activity was detected by the release of the dye of synthetic substrate 4-nitrophenyl-β-D-xylanopyranoside. To detect enzyme activity in the well 1 volume of culture liquid, then 4 volumes of buffer (50 mM Tris-HCl, 1 mM CaCl_2_, pH 9.0), and 5 volumes of 100 mg 4-nitrophenyl-β-D-xylanopyranoside solution was added to 10 mL 100 mM PBS. Cultivation was carried out at 37 °C. The course of the reaction was detected by Epoch (Agilent Technologies (formerly BioTek Instruments), Santa Clara, CA, USA) well spectrometer. Evaluation was performed every 10 min at 405 nm. Supernatant of *K. phaffii* T07 strain without the inserted xylanase gene cell culture was used as a negative control. 

### 4.5. Enzyme Cultivation in Bioreactor

Cultivation of xAor xylanase was performed using ProLab (GPC, La Rochelle, France) bioreactor. Respective *K. phaffii* clone was sewed on the agarized yeast extract peptone dextrose media to obtain separate colonies for 48 h, and then inoculated in 5 mL yeast extract peptone dextrose media with 200 µg/mL zeocin, and incubated at 30 °C all night. Then all-night culture was inoculated in yeast nitrogen base media in 1:100 volume (5 L) and cultivated at 250 rpm in shaker at 30 °C for 48 h.

Primary culture was aseptically inoculated in a bioreactor, with the salt media consisting of 32.5 g/l glycerin, 9.38 g/L (NH_4_)_2_SO_4_, 1.88 g/L CaSO_4_∙2H_2_O, 0.94 g/L NaCl, 3.75 g/L MgSO_4_∙7H_2_O, and 3.75 g/L KH_2_PO_4_. Bioreactor was previously sterilized for 45 min at 121 °C.

At the start of cultivation, temperature was 30 °C with the constant air flow 3 L/min and initial mixer velocity 400 rpm. Concentration of dissolved oxygen was kept at >20% by gradual mixer velocity increasing up to 1200 rpm, and media pH—5.8–6.0, using 4 M NaOH solution. Before the inoculation, 2.5 mL/L microelements and 2.5 mL/L vitamins were added (Table 1).

### 4.6. Enzyme Purification from Culture Liquid

Culture liquid was separated from cells and other suspended materials by centrifugation at 4000 rpm for 10 min. The supernatant was purified and concentrated with tangential ultrafiltration, using SartoJet (Sartorius, Göttingen, Germany) with 10 kDa pore filters. All the reaction were carried out at 5 °C. 

Concentrate was frozen at −70 °C and freeze-dried (Labconco, Kansas City, MO, USA). Obtained half-product was dissolved in 10 mM sodium phosphate buffer (pH 7.5), purified from chromogenic additives by gel-filtration using Sephadex G-25 (Sigma-Aldrich, Schnelldorf, Germany). Elution was carried out with 10 mM sodium phosphate buffer (pH 7.5). Protein purification was performed using ion-exchange chromatography on the column with anionite DEAE-Sepharose 6HF (Biotoolomics, Consett, Great Britain). Column was washed with 10 mM sodium phosphate buffer (pH 7.5), target protein was eluted with linear gradient 0.0–0.5 M NaCl in starting buffer. Purity grade in fractions with the highest enzyme activity were analyzed by electrophoresis in poly acryl-amide gel. Fractions, containing minimal amount of additional proteins were combined, desalted, and concentrated, using centrifugal filters with 10 kDa pore size and freeze-dried.

### 4.7. Enzyme Activity Determination at Various pH and Temperatures

Xylanase activity was determined by GOST 31488-2012 “Enzyme preparations. Methods of xylanase enzyme activity determination” using 1% oat xylane substrate [23]. To do this, 2 g of oat xylane was dissolved in 50 mL purified water with constant mixing at room temperature for 30–60 min. Next, solution was centrifuged at 4000 rpm for 10 min at 5 °C. Volume of obtained supernatant was brought up to 100 mL with purified water or 50 mM buffer solution.

To evaluate the operating pH range, the following buffers were used: for pH 2.5–3.5—50 mM Gly-HCl buffer; for pH 4.0–5.5—50 mM sodium acetate buffer; pH 6.0–7.0—50 mM potassium phosphate buffer; for pH 7.5–11.0—50 mM Tris-Gly buffer. Samples with xylanase activity were dissolved in 50 mM buffer.

The assay was performed as follows: 250 µL substrate in the respective buffer was heated for 5 min at 50 °C. Next, to the preheated substrate solution equal volume of enzyme solution, while in respective negative control—equal volume of buffer solution. Enzyme reaction was carried out for 10 min a t 50 °C. The reaction was interrupted by the addition of 750 µL 1% dinitrosalicylic acid. All samples were incubated for 10 min at a boiling water bath until coloring development. Optical density was measured at 546 nm.

Temperature operating range of xAor xylanase was performed as described above with following modifications: enzyme reaction was carried out at pH = 7.5 in all cases, but incubation temperature varied. 

### 4.8. Electrophoretic Analysis

To perform an electrophoretic analysis, lyophilized sample was dissolved in 1 mL 62.5 mM Tris-HCl pH 6.8 with 2% phenylmethylsulfonyl fluoride and 1% sodium dodecyl sulfate (DSN), and treated with ultrasound on ice for 3 min 2 s impulses with 2 s pauses, using ultrasound probe with 91 Watt capacity (Cole-Parmer Instrument, Vernon Hills, IL, USA). Next, sample was mixed with application buffer, consisted of 62.5 mM Tris-HCl pH 6.8, 25% glycerin, 2% DSN, 0.01% bromophenol blue, and 5% β-mercapthoethanol in 1:2 volume, and incubated at 100 °C for 5 min. 

Sample was concentrated in 4% PAAG-DSN at 15 mA and separated in acrylamide/bis-acrylamide gel with 0.1% DSN, 0.38 mM Tris-HCl with pH 8.8, 0.05% TEIUD, 0.05% ammonium persulfate at 25 mA in electrophoretic chamber Mini-PROTEAN^®^ Tetra Cell (Bio-Rad, Hercules, CA, USA). Gel was colored with SYPRO™ Ruby (Invitrogen, Waltham, MA, USA), and visualized by gel-documenting system VersaDoc MP4000 (Bio-Rad, Hercules, CA, USA).

### 4.9. Mass-Spectrometry

Lyophilized sample of culture liquid was dissolved in 100 mM NH_4_HCO_3_ to the concentration of 5 mg/mL. Next, sample was with 20 mM TCEP, 40 mM chloroacetamide, 100 mM NH_4_HCO_3_, and 0.1% sodium deoxycholate buffer for 5 min at +4 °C at 1:20 volume, 5 min at 90 °C, and 20 min at RT in the dark. Tripsin was added at 1:100 volume and incubated for 16 h at 37 °C. Reaction was disrupted by the addition of 1% trifluoromethyl ketone. Samples were emulsified with the ethylacetate equal volume thrice for 5 min with organic phase removal. For the third extraction ethilacetate with 1% TFA was used. Sample, equal to 20 µg of protein, was purified, using StageTips, according to Rappsilber et al. [24]. Sample was concentrated with C18 cartridge by solid phase extraction, and then recovered in the first step of UHPLC gradient. Next, samples were dried with rotation evaporator, stored at –20 °C, and dissolved in 20% ACN with 0.1% TFA to obtain protein concentration of 1 µg/µL. Sample, mixed with 20 mg/mL 2,5-dihydroxybenzoic acid, 0.1% TFA, and 70% ACN, was then applied to the mass-spectrometer target.

Identification of MS2 was carried out using UltraFlex III MALDI tandem time-of-flight (Bruker, Munich, Germany), MS/MS digest—LTQ-Orbitrap-XL-ETD (Thermo Scientific, Waltham, MA, USA). For the detection from optimized theoretical protein sequence Mascot software with Swissprot and ICIG_enzymes (database, compiled from theoretical protein sequences) databases were used.

## 5. Conclusions

Xylanases have applications in food and beverages industry, animal nutrition, wood pulp bioleaching, and papermaking. Taking into consideration the wide working range of pH and temperatures enzyme activity, and the ability to be produced in a bioreactor, the authors suggest that xAor xylanase may have suitable industrial application. Authors suggest that with high activity in the culture liquid xylanase, xAor may be used for industrial applications without being purified or concentrated. In concussion, protocol for soluble, secreted xylanase suitable for industrial scale production was obtained.

## Figures and Tables

**Table 1:** Amount of added chemicals in 5 L of media.

Chemicals	Amount per 1 L
CuSO_4_∙5H_2_O	3 g
NaI	0.4 g
MnSO_4_	2 g
Na_2_MoO_4_∙2H_2_O	1 g
H_3_BO_3_	0.1 g
CoCl_2_∙6H_2_O	0.5 g
FeSO_4_∙7 H_2_O	33 g
H_2_SO_4_	5 mL
ZnSO_4_∙7H_2_O	5 g
vitamin H	0.05 g
vitamin B_5_	0.2 g
vitamin B_9_	0.01 g
*myo*-inositol	1 g
vitamin PP	0.2 g
*para*-aminobenzoic acid	0.1 g
vitamin B_6_	0.2 g
vitamin B_2_	0.1 g
vitamin B_1_	0.2 g

## Data Availability

The authors confirm that the data supporting the findings of this study are available within the article.

## Appendix A

The escrow notes and passports on the deposition of the strain *Komagataella phaffii* T07 (Accession Number #Y-4936) and *Komagataella phaffii* Y-5026 deposited by EFKO (Efirnoe Joint-Stock Company, OOO IC Biryuch-NT) is presented https://vkpm.genetika.ru/ (accessed on 2 August 2022).
